# Beta-2 microglobulin is important for disease progression in a murine model for amyotrophic lateral sclerosis

**DOI:** 10.3389/fncel.2013.00249

**Published:** 2013-12-10

**Authors:** Kim A. Staats, Susann Schönefeldt, Marike Van Rillaer, Annelies Van Hoecke, Philip Van Damme, Wim Robberecht, Adrian Liston, Ludo Van Den Bosch

**Affiliations:** ^1^KU Leuven Laboratory of Neurobiology and Leuven Research Institute for Neuroscience and Disease (LIND)Leuven, Belgium; ^2^VIB Vesalius Research Center, KU LeuvenLeuven, Belgium; ^3^VIB Autoimmune Genetics Laboratory, KU LeuvenLeuven, Belgium; ^4^Department of Microbiology and Immunology, University of LeuvenLeuven, Belgium; ^5^Neurology, University Hospitals LeuvenLeuven, Belgium

**Keywords:** beta-2 microglobulin, amyotrophic lateral sclerosis, motor neuron, neurodegeneration, motor neuron disease

## Abstract

Beta-2 microglobulin (*β*2*m*) is an essential component of the major histocompatibility complex (MHC) class I proteins and in the nervous system *β*2*m* is predominantly expressed in motor neurons. As *β*2*m* can promote nerve regeneration, we investigated its potential role in amyotrophic lateral sclerosis (ALS) by investigating its expression level as well as the effect of genetically removing *β*2*m* on the disease process in mutant superoxide dismutase 1 (*SOD1*^*G*93*A*^) mice, a model of ALS. We observed a strong upregulation of *β*2*m* in motor neurons during the disease process and ubiquitous removal of *β*2*m* dramatically shortens the disease duration indicating that *β*2*m* plays an essential and positive role during the disease process. We hypothesize that *β*2*m* contributes to plasticity that is essential for muscle reinnervation. Absence of this plasticity will lead to faster muscle denervation and counteracting this process could be a relevant therapeutic target.

## Introduction

Major histocompatibility complex (MHC) class I proteins were originally discovered based on their critical role in the immune system, however immune-independent functions in the nervous system have recently been identified (Huh et al., 2000; Elmer and Mcallister, 2012). Beta-2 microglobulin (*β*2*m*) is an essential component of MHC class I molecules, being required for expression of all MHC class I on the cell surface. Within the central nervous system *β*2*m* has a predominantly motor neuronal expression pattern (Linda et al., 1998, 1999; Thams et al., 2009). This protein is therefore a candidate to contribute to the selective vulnerability of such motor neurons during amyotrophic lateral sclerosis (ALS).

ALS is a progressive neurodegenerative disease, characterized by the selective loss of motor neurons and the denervation of muscle fibers, resulting in muscle weakness and paralysis. In Europe, the disease has an annual incidence of 2.7 cases per 100,000 people (Logroscino et al., 2010) and the disease duration post-diagnosis is 3–5 years. In 10% of patients, ALS is a familial disease and 20% of these familial ALS patients contain mutations in the gene encoding superoxide dismutase 1 (*SOD1*). Based on these mutations, ALS rodent models have been generated that predictably mimic the patient disease process (Julien and Kriz, 2006). As the disease progression is indistinguishable between familial and sporadic ALS, common disease mechanisms are predicted. One of these mechanisms is decreased (peripheral) neuronal plasticity that can influence the ability of neuronal networks to compensate for a loss of (motor) neurons in the network. *β*2*m* is expressed in motor neurons in the lumbar spinal cord (Linda et al., 1999) as well as in motor axons (Thams et al., 2009). Additionally, *β*2*m* promotes recovery after axotomy (Linda et al., 1998; Oliveira et al., 2004) and sciatic nerve crush (Oliveira et al., 2004), which implies that it may be of importance in ALS too.

In this study, we investigated the role of *β*2*m* in ALS mice. To this end, we assessed the gene expression of *β*2*m* and interbred mice genetically lacking *β*2*m* with *SOD1*^*G*93*A*^ mice and assessed survival and disease pathology.

## Materials and methods

### Animal experiments

Mice overexpressing human wild-type *SOD1* (*SOD1^WT^*) or human *SOD1*^*G*93*A*^ and *β*2*m* knockout mice were purchased from The Jackson Laboratories (Bar Harbor, USA) and maintained on a C57BL/6 background. The *SOD1*^*G*93*A*^ and *β*2*m* knockout were interbred allowing for approx. 50% of the mice to be littermate controlled in this study. Chow and water were provided *ad libitum* and mice were housed in the specific pathogen free animal facility of the KU Leuven under standard conditions according to the guidelines of the KU Leuven. End stage was defined as the age at which mice could no longer right themselves within 30 s when placed on their back. End stage is used as a measurement of survival and is the condition at which mice are euthanized to prevent further suffering. Disease onset was defined as the age at which mouse weight dropped below 90% of the average day 90–105 weight. The animal caretakers and scientists were blinded to the genotypes of the mice when assessing “end stage”. All animal experiments were performed with the approval of the Animal Ethical Committee of KU Leuven (020/2010).

### Laser dissection microscopy

Murine spinal cords were snap-frozen in Tissue-Tec (Sakura Finetek Europe, Alphen aan de Rijn, The Netherlands) to make cryostat sections of 20-μm thickness. Then, cresyl violet–stained motor neurons, located in the ventral horn of the lumbar spinal cord, were collected on membrane slides 1.0 PEN (Carl Zeiss AG, Oberkochen, Germany), using dissection by a laser-dissection microscope (Carl Zeiss AG) and capturing in Adhesive Cap 500 opaque (Carl Zeiss AG). Only motor neurons in which the nucleus was visible and with soma area > 250 μm^2^, were collected. At least 1,500 motor neurons were dissected for each animal.

### Quantitative PCR

Isolation of mRNA was performed using the TriPure (Roche, Basel, Switzerland) method and the RNeasy kit (Qiagen, Venlo, The Netherlands). Reverse transcriptase polymerase chain reaction (PCR) used random hexamers (Life Technologies, Carlsbad, USA) and Moloney Murine Leukemia Virus Reverse Transcriptase (MMLV RT; Invitrogen, Carlsbad, USA). Quantitative PCR (qPCR) was performed with the StepOnePlus (Life Technologies) and TaqMan Universal PCR Master Mix (Life Technologies). Gene expression assays were purchased from Life Technologies and IDT DNA (Coralville, USA): *gapdh* (Mm.PT.39a.1), *β*2*m* (Mm00437762_m1) and *cd8b1* (Mm.PT49a.10182911). For this analysis, presymptomatic tissue was collected at 90 days of age and symptomatic at 120 days of age. The scientist performing the qPCR was blinded to the genotypes of the samples.

### Nissl staining

To visualize neurons, Nissl staining was performed on 4% formaldehyde fixed spinal cords sections. Sections were briefly immersed in a cresyl violet solution and subsequently in a 70% ethanol with 10% acetic acid. Slides were dehydrated by an increased ethanol concentration series and mounted with PerTex^®^ (Histolab AB, Goteborg, Sweden). Images were collected by Zeiss Axio Imager M1 microscope (Carl Zeiss AG) with AxioCam Mrc5 camera (Carl Zeiss AG). The number of (motor) neurons was quantified by measurement of the soma area as visualized by cresyl violet staining in ImageJ (National Institute of Health) on multiple 40 μm thick sections in the ventral horn of the lumbar spinal cord. Characterisation of motor neurons occurred as previously (Fischer et al., 2004) of at least 5–10 ventral horns of the lumbar spinal cord of 2–4 mice per group. The scientist performing the Nissl staining and neuron quantification was blinded to the genotypes of the samples.

### Immunohistochemistry

Mice were transcardially perfused with phosphate buffered saline (PBS) and subsequently with 4% formaldehyde. Spinal cords were post-fixed with 4% formaldehyde overnight at 4°C and transferred to 30% sucrose for an additional night. After snap freezing, tissue was sectioned by cryostat at 40 μm thickness and stained with a polyclonal antibody directed against ubiquitin (Dako, Glostrup, Denmark). Images were collected by Zeiss Axio Imager M1 microscope (Carl Zeiss AG) with AxioCam Mrc5 camera (Carl Zeiss AG). Ubiquitin immunopositive aggregates were counted per ventral horn using ImageJ of 2–6 ventral horns of the lumbar spinal cord of 2–4 mice per group and presented as the average of the number of aggregates per ventral horn. The scientist performing the immunohistochemistry and aggregate quantification was blinded to the genotypes of the samples.

### Statistical analysis

Analysis was performed with the statistical software package Prism Origin (GraphPad Software, La Jolla, USA). Survival was analyzed by Log-Rank testing. Differences between two groups were analyzed using a Student’s *t*-test. Differences between more than two groups were analyzed by ANOVA with Bonferroni correction for multiple testing. Significance was assumed at *p* < 0.05. Error bars represent the standard deviation.

## Results

**Figure 1:**
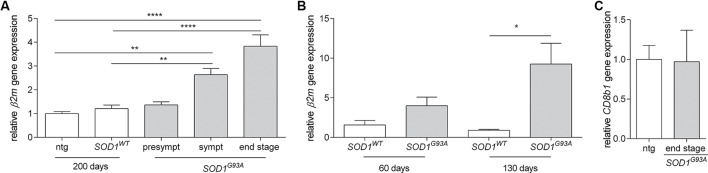
**Increased *β*2*m* gene expression in ALS mice**. **(A)** Relative *β*2*m* gene expression in spinal cord of non-transgenic (ntg, *n* = 6) and *SOD1^WT^* controls (*n* = 6) compared to presymtomatic (presympt, *n* = 6), symptomatic (sympt, *n* = 6) and end stage (*n* = 6) *SOD1*^*G*93*A*^ mice (ANOVA, Bonferroni post hoc). **(B)** Relative *β*2*m* gene expression in neurons isolated by laser dissection microscopy from the spinal cord of *SOD1^WT^* controls at 60 days (*n* = 2) and 130 days of age (*n* = 3) compared to neurons from *SOD1*^*G*93*A*^ mice at 60 days (*n* = 3) and 130 days of age (*n* = 3; Student’s *t*-test). **(C)** Relative *CD8b1* gene expression in the spinal cord of 150 day old non-transgenic mice (ntg, *n* = 5) and end stage (*n* = 5) *SOD1*^*G*93*A*^ mice. *p < 0.05, **p < 0.01, ****p < 0.0001.

To assess the potential for *β*2*m* to have a functional role in ALS pathogenesis, we assessed gene expression in the spinal cords of non-transgenic, *SOD1^WT^* and *SOD1*^*G*93*A*^ mice. We observed a strong increase in *β*2*m* gene expression during disease progression in *SOD1*^*G*93*A*^ mice (Figure 1A). This increase is at least partly due to the increased neuron-specific gene expression of *β*2*m*, as a greater level of upregulation (10-fold) was identified by qPCR on neurons from *SOD1*^*G*93*A*^ mice compared to neurons from *SOD1*^WT^ mice isolated by laser dissection microscopy (Figure 1B). As we do not observe an increase of CD8^+^ T cells in the spinal cord of end stage *SOD1*^*G*93*A*^ mice, as assessed by the gene expression analysis of *CD8b1* (Figure 1C), these data indicate potential for a neuronal role for *β*2*m* in ALS.

**Figure 2:**
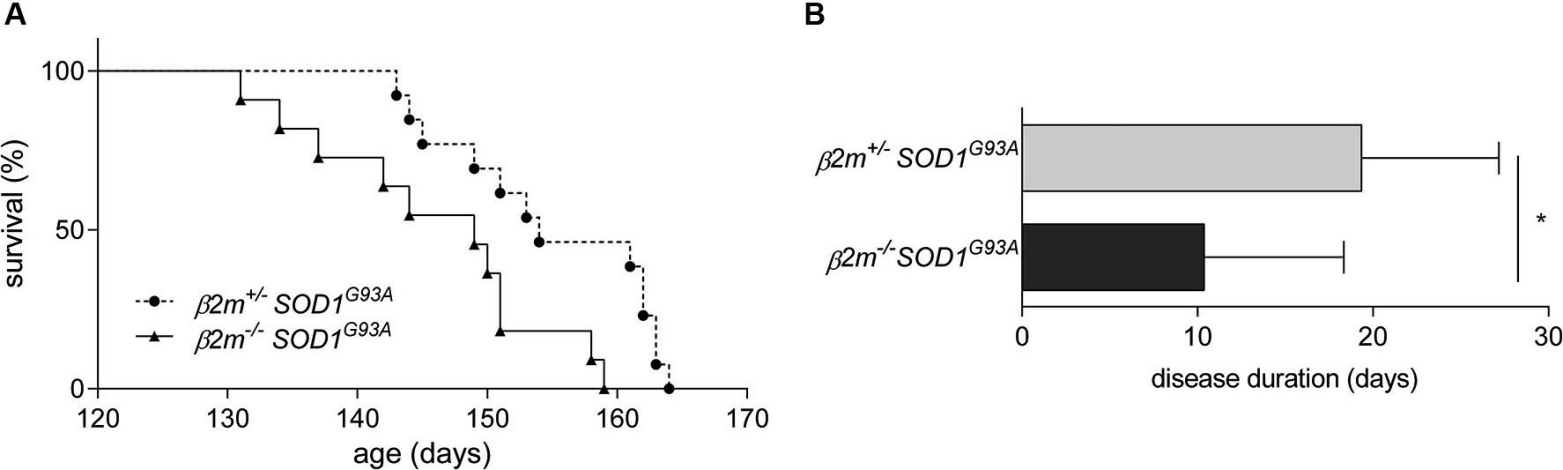
**Decreased survival in *β*2*m* knockout ALS mice**. **(A)** Survival analysis of *β*2*m*^+/–^ *SOD1*^*G*93*A*^ (*n* = 13, 154.9 ± 7.7 days) and *β*2*m*^−/–^ *SOD1*^*G*93*A*^ mice (*n* = 11, 146.0 ± 8.8 days; Log-Rank *p* = 0.009). **(B)** Disease duration of *β*2*m*^+/–^ *SOD1*^*G*93*A*^ (*n* = 9) and *β*2*m*^−/–^ *SOD1*^*G*93*A*^ mice (*n* = 8). *p < 0.05.

To determine whether *β*2*m* has an effect in ALS, we interbred *β*2*m*^−/–^ mice with *SOD1*^*G*93*A*^ mice and assessed disease progression in *β*2*m*^−/–^ *SOD1*^*G*93*A*^, *β*2*m*^+/–^ *SOD1*^*G*93*A*^ and *β*2*m*^+/+^ *SOD1*^*G*93*A*^ mice. The survival of *β*2*m*^+/–^ *SOD1*^*G*93*A*^ mice did not differ from *β*2*m*^+/+^ *SOD1*^*G*93*A*^ littermates (data not shown). The complete genetic ablation of *β*2*m* did not affect onset of disease (data not shown), but significantly decreased average survival of *SOD1*^*G*93*A*^ mice by 8.9 days (Figure 2A) and reduced disease duration by approximately 50% (Figure 2B). The decrease of survival in *β*2*m*^−/–^ *SOD1*^*G*93*A*^ mice demonstrated a protective role for *β*2*m* in ALS mice.

**Figure 3:**
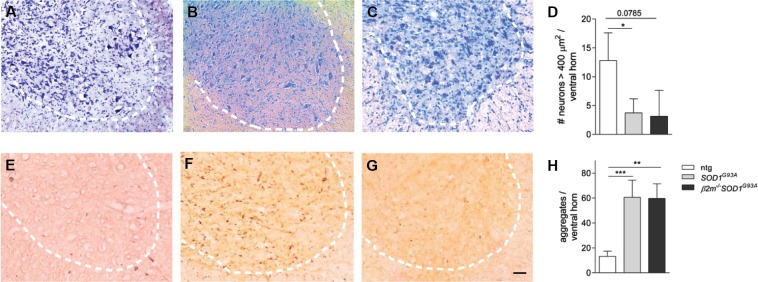
**Unaltered end stage pathology in *β*2*m*-deficient *SOD1*^*G*93*A*^ mice**. Ventral horns of spinal cord sections of non-transgenic (ntg, *n* = 4; **A** and **E**) and *SOD1*^*G*93*A*^ (*n* = 4; **B** and **F**) and *β*2*m*^−/–^ *SOD1*^*G*93*A*^ (*n* = 2; **C** and **G**) *SOD1*^*G*93*A*^ mice. Nissl staining was used to visualize the (motor) neurons **(A–C)**. The number of > 250 μm^2^ neurons and > 400 μm^2^ motor neurons per ventral horn are quantified in **(D)**. Ubiquitin immunoreactivity in spinal cord sections of non-transgenic, *SOD1*^*G*93*A*^ and *β*2*m*^−/–^ *SOD1*^*G*93*A*^ mice are quantified in **(H)** Dashed lines delineate the ventral horn. Scale bar = 100 µm. *p < 0.05, **p < 0.01, ***p < 0.001.

To assess whether genetic ablation of *β*2*m* alters pathology of *SOD1*^*G*93*A*^ mice, we analyzed pathology in the spinal cords of end stage mice. Decreased numbers of motor neurons were observed in the end stage spinal cord of *SOD1*^*G*93*A*^ and *β*2*m*^−/–^ *SOD1*^*G*93*A*^ mice (Figures 3A–C, quantified in Figure 3D), as were increased ubiquitin-positive aggregates (Figures 3E–G, quantified in Figure 3H). No differences were observed between the end stage pathology of *SOD1*^*G*93*A*^ and *β*2*m*^−/–^ *SOD1*^*G*93*A*^ mice for motor neurons (Figures 3B, C) or ubiquitin immunoreactivity (Figures 3F, G). This shows that end stage *β*2*m*^−/–^ *SOD1*^*G*93*A*^ mice show the same extent of motor neuron loss and aggregate formation as end stage *SOD1*^*G*93*A*^ mice, although disease progression is faster.

## Discussion

Here we show that *β*2*m* is important in ALS mouse survival and that it is upregulated during disease in the spinal cord and by motor neurons. Upregulation of *β*2*m* in neuronal tissues has been reported previously when comparing spinal cord (Edstrom et al., 2004) and brain (VanGuilder Starkey et al., 2012) expression of aged rats to adult controls and in the spinal cord of axotomised rats (Maehlen et al., 1988; Olsson et al., 1989; Linda et al., 1998), which may suggest that stressed neurons increase *β*2*m* gene expression to increase plasticity. This concept fits well with reports of the role of *β*2*m* in neurons during development and plasticity (Huh et al., 2000; Bilousova et al., 2012), of the hippocampus and visual system (Huh et al., 2000) but not of the cerebellum (Letellier et al., 2008), and the delayed or impaired recovery of *β*2*m* knockout mice post axotomy (Linda et al., 1998; Oliveira et al., 2004) and sciatic nerve crush (Oliveira et al., 2004).

Impaired (peripheral) plasticity by *β*2*m* knockout may explain the decrease in survival detected in ALS mice in this study, as increased plasticity is protective in ALS mice and rats (Van Hoecke et al., 2012). A number of plasticity-promoting genetic or pharmacological strategies have proven successful in the past in ALS models, such as EphA4 knockdown and inhibition (Van Hoecke et al., 2012), and vascular endothelial growth factor (VEGF) administration in ALS rodents (Storkebaum et al., 2005).

With the use of a ubiquitous *β*2*m* knockout mouse we cannot exclude that the decrease of survival of ALS mice lacking *β*2*m* may be due to the effect of removing *β*2*m* in the immune system. *β*2*m* is necessary for the differentiation of CD8^+^ T cells and natural killer T (NKT) cells (Koller et al., 1990). The role of these cell types is not yet fully understood in ALS, as varying results are obtained for ALS mouse survival when mature lymphocytes are not present (Beers et al., 2008; Tada et al., 2011). Additionally, NKT cells may be associated to ALS disease pathology as impairments in NKT cells are reported in ALS mice (Finkelstein et al., 2011). That being said, qPCR analysis of CD8^+^ T cells does not suggest a role for these cells as *CD8b1* gene expression is not increased in ALS spinal cords. Additionally, a role for CD8^+^ T cells may be predicted to be detrimental in contrast to our data demonstrating a detrimental role for *β*2*m* upon removal in ALS mice. Interestingly, *β*2*m* has been assessed previously as a biomarker in cerebrospinal fluid or venous blood samples from ALS patients, but with variable results (Brettschneider et al., 2008; Mitchell et al., 2009; Baciu et al., 2012).

The mechanism to which *β*2*m* contributes to neuronal plasticity is not fully understood, though it is proposed that it may be through paired-immunoglobulin like receptor-B (PirB) (VanGuilder Starkey et al., 2012). This receptor is located on axons, dendrites and neuronal somata (VanGuilder Starkey et al., 2012) and could thus easily facilitate plasticity. Additionally, *β*2*m* is localized at synapses and in synaptosomes (Shatz, 2009). Alternatively, *β*2*m* and PirB are associated with decreased plasticity and recovery in other neurodegenerative conditions such as stroke (Adelson et al., 2012), experimental autoimmune encephalomyelitis (EAE; Denic et al., 2012) and ischemia (Wang et al., 2012). These paradigms are largely affected by the immune system and the beneficial effect of *β*2*m* or PirB in these models may be due to the role of the immune system. This notion is supported by work by Linker et al. that show that reconstitution of CD8^+^ cells in *β*2*m* knockout mice delays the effect of EAE compared to *β*2*m* knockout littermates (Linker et al., 2005).

In conclusion, this work shows the detrimental effect of *β*2*m* knockout in ALS and identifies *β*2*m* signaling as a potential new direction for the development of therapeutic strategies counteracting ALS.

## Author contributions

Kim A. Staats and Susann Schönefeldt performed the murine behavioral analyses. Kim A. Staats and Marike Van Rillaer conducted the staining experiments. Kim A. Staats and Annelies Van Hoecke analyzed gene expression of motor neurons excised by laser dissection microscopy. Philip Van Damme, Wim Robberecht, Adrian Liston and Ludo Van Den Bosch supervised and designed the experiments. Kim A. Staats, Adrian Liston and Ludo Van Den Bosch wrote the manuscript. All authors approved the final version of the manuscript.

## Conflict of interest statement

The authors declare that the research was conducted in the absence of any commercial or financial relationships that could be construed as a potential conflict of interest.
